# A Rare Initial Presentation of Aortic Intramural Hematoma: A Case Report and Literature Review

**DOI:** 10.7759/cureus.32947

**Published:** 2022-12-26

**Authors:** Kyle Coombes, Kayvon Moin, Mohammad A Ahmed-Khan, Jonathan Vargas

**Affiliations:** 1 Medical School, American University of the Caribbean, Cupecoy, SXM; 2 Medicine, CMH Lahore Medical College and Institute of Dentistry, Lahore, PAK; 3 Internal Medicine, Danbury Hospital, Danbury, USA

**Keywords:** aortic dissection, stanford classification, aortic intramural hematoma, acute aortic syndrome, cardiology

## Abstract

Aortic intramural hematoma (AIH) is a life-threatening emergency that involves aortic wall integrity and is characterized by either a direct rupture of the vasa vasorum or spontaneous bleeding of an arterial plaque located in the tunica media of the aortic wall. A notable difference between AIH and acute aortic dissection is the absence of an intimal flap, a finding discernable on computed tomography angiography (CTA). Follow-up imaging allows for the monitoring of disease progression or early findings of impending complications. While some patients may require surgical intervention, medical management with blood pressure control remains the mainstay in treatment. Our case describes a patient who was found to be in cardiac arrest secondary to ventricular fibrillation and was then found to have presumed Stanford Type A aortic dissection on CTA. After reviewing the scans, the diagnosis was reclassified to AIH due to the absence of an intimal flap, the patient was then managed medically for AIH with antihypertensive medications.

## Introduction

Aortic intramural hematoma (AIH) is a life-threatening emergency that involves aortic wall integrity, making up one-third of acute aortic syndrome (AAS) cases [1]. AAS is an umbrella term that encompasses several aortic pathologies including aortic dissection (AD), penetrating atherosclerotic ulcer (PAS), AIH, and iatrogenic traumatic aortic injury [1]. 

While the aorta comprises three distinct layers, the Tunica Intima, Media, and Adventitia, the intimal layer, or innermost, is the most frequently damaged area in patients with poorly controlled blood pressure, a major risk factor for AAS [2,3]. This is due to the wall-shearing effects of uncontrolled blood pressure that disrupts and damages the network of elastic fibers within the intimal layer leading to fragmentation and tears [4]. However, in AIH, the intimal layer remains intact, a key characteristic that differentiates it from AD [1].

## Case presentation

An 81-year-old male, with a pertinent past medical history of atrial fibrillation on rivaroxaban, heart failure with a reduced ejection fraction (HFrEF) of 30%, thoracic aortic aneurysm, prior myocardial infarction at the age of 42, and hypertension was brought into the emergency department (ED) after collapsing at the supermarket. Emergency medical services (EMS) arrived, and the patient was found to be apneic with no palpable pulse due to cardiac arrest secondary to ventricular fibrillation. CPR was taken over by the EMS and the patient received a total of three shocks, three push doses of epinephrine, and two boluses (150 mg and 300 mg) of amiodarone on the way to the ED. After the three shocks were completed, the patient had a subsequent return of spontaneous circulation (ROSC), spontaneous respirations, and heart rate in the 50s. Upon arrival at the ED, the patient was unresponsive to verbal stimuli with a positive gag reflex and was immediately intubated. Bedside systolic blood pressures were in the 90s and the heart rate was ranging between 40 to 60 beats per minute; a lidocaine drip at the rate of 1 mg/min was initiated on the patient for arrhythmia management. Shortly after arrival at the ED, the patient developed agitation and hypertensive urgency with systolic blood pressures up to the 190s-200s and was placed on a nicardipine drip. 

A 12-lead electrocardiogram (ECG) showed atrial fibrillation with underlying right bundle branch block (RBBB) and QT prolongation at about 500 ms. CT of the brain/head showed a large frontal scalp hematoma with laceration and CT of the spine showed no trauma-related changes. A bedside point-of-care ultrasound (POCUS) was performed and revealed a dilated aorta. Due to the patient’s history of thoracic aortic aneurysm, a CT angiography (CTA) of the chest and abdomen was ordered. Initial evaluations of the imaging suggested a Stanford type A aortic dissection extending from the aortic root, involving the arch and descending aorta, and up to the level of the diaphragmatic crura. 

**Figure 1:**
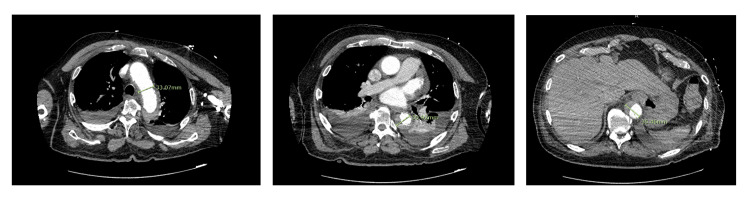
(Left to right) CT angiography with axial cut showcasing the patient’s aortic intramural hematoma (demarcated in green) extending essentially from the aortic root to just cranial to the renal arteries although without extent into any major branch vessels.

The patient was transferred to the intensive care unit (ICU) and was only managed medically with labetalol, lidocaine, and propofol drip as the family was unsure of proceeding with surgical management. On the second day in the ICU, the patient was successfully extubated and continuously managed medically. The cardiothoracic surgery team following the case re-examined the CTA of the chest and abdomen that was performed and determined that there was no evidence of an intimal flap, suggesting a more appropriate diagnosis of aortic intramural hematoma (Figure 1). The patient continued to be managed non-surgically in the ICU with IV antihypertensive medication with a goal systolic blood pressure of 120 mmHg or less. Serial CTA scans were performed, which showed the hematoma to be stable and slightly improving. After adequate blood pressure control, the patient was transferred to the general medicine floor and managed with oral antihypertensive medications until discharge.

## Discussion

AIH is a vascular disorder involving the medial layer of the aorta that is thought to be secondary to the rupture of the vasa vasorum or aortic blood supply [5-7]. Whether direct rupture of the vasa vasorum or spontaneous bleeding of microvessels in an arterial plaque, the outcome is the same [6]. Blood accumulates within the medial layer of the aorta wall without any intimal damage present, a distinguishing feature that separates AIH from AD [7]. While some cases may result in spontaneous resorption, others may progress to more severe and life-threatening complications (eg, AD, aneurysm formation, myocardial infarction, cardiac tamponade, aortic regurgitation, and death) [8].

The DeBakey and Stanford system are two classifying methods used to describe aortic syndromes and define their location. The Stanford system subdivides location based on proximal (type-A) or distal (type-B) relationship to the left subclavian artery [9]. The DeBakey classification labels pathologies involving the ascending aorta and aortic arch as Type-1, ascending aorta as Type-2, and the descending aorta to the diaphragm as Type-3 [9].

Timely imaging is vital for diagnosis, especially in hemodynamically unstable patients [10]. CTA is the gold standard for diagnosing aortic syndromes, however, MRI and echocardiography also have utility [11]. MRI is beneficial in confirming uncertain cases due to its ability to distinctively define the vascular wall, but its longer processing time makes it unsuitable for acute cases [8]. Transthoracic echocardiography (TTE) has the advantage that it can be performed at the bedside and provide real-time results, making it a popular choice of imaging in most cases [12,1]. This method of imaging allows clinicians to quickly assess aortic wall thickness and identify any echolucent, crescent-shaped zones within the aortic wall [12,1]. Characteristically, a >7 mm aortic wall thickness is diagnostic for AIH, yet >5 mm has been accepted in the clinical setting [8,1].

Patients must be followed serially with imaging due to the unpredictable nature of AIH. In some cases, the hematoma can propagate within the false lumen of the aortic wall and interfere with the heart’s physiologic properties, as described in Gheshlaghi et al.’s report [10]. Recent reviews have also found worsening outcomes to be associated with radiologic findings of proximal migration of the hematoma, hematoma thickness >11 mm, aortic diameter >50 mm, progressive aortic enlargement, ulcer-like projections, focal intimal disruption, and poor blood pressure control [1,13].

Blood pressure control with beta-adrenergic receptor blockade in combination with calcium channel blockers or sodium nitroprusside, to avoid reflex tachycardia, remains the first-line intervention in patients with AAS [13]. While the management of Stanford Type-A AIH is debated within the current literature, recent reviews have recorded better outcomes in those managed surgically versus medically [14]. On the other hand, Type-B AIH is regularly managed medically with hemodynamic control [15].

## Conclusions

AIH is a potentially fatal emergency that is more commonly seen in older individuals with poor blood pressure control. Diagnosis resides on a combination of patient presentation, clinical history, laboratory blood work, and imaging techniques. These components must be taken into consideration to differentiate from similar diagnoses, such as aortic dissection. Serial imaging is important throughout follow-up to evaluate for possible future complications.
